# Prevention of cardiovascular events in heart failure with mildly reduced or preserved ejection fraction: a comprehensive network meta-analysis of eight randomized controlled trials using reconstructed individual patient’s data

**DOI:** 10.1016/j.eclinm.2025.103506

**Published:** 2025-09-12

**Authors:** Alhassane Diallo, Miguel Carlos-Bolumbu, Philippe Duc, Florence Galtier

**Affiliations:** a Department of Public Health, University Gamal Abdel Nasser of Conakry, Conakry, Guinea; b Emergency Resuscitation Centre Hospitalier Sud Essonnes CHSE, Paris, France; c Service de Réadaptation Cardiovasculaire Groupe Hospitalier Paris Saint Joseph, Paris, France; d INSERM, Clinical Investigation Center 1411 & Department of Endocrinology, Diabetes, and Nutrition, Montpellier University Hospital, Univ Montpellier, 80 Avenue Augustin Fliche, Montpellier Cedex 5 34295, France

**Keywords:** Additive network meta-analysis, Heart failure event, HFmEF, HFpEF, Reconstructed individual patient data

## Abstract

**Background:**

Cardiometabolic therapies such as mineralocorticoid receptor antagonists (MRAs), sodium glucose cotransporter 2 inhibitors (SGLT2i), and glucagon-like peptide 1 receptor agonists (GLP-1 RA) reduce clinical heart failure events in patients with heart failure with mildly reduced or preserved ejection fraction (HFpEF). However, evidence for the benefit of one strategy or their combination is not well established. To optimize guidelines in this population, evidence of the most effective therapeutic options or strategies is needed.

**Methods:**

The present network meta-analysis used reconstructed individual patient data from published Kaplan–Meier curves of cardiometabolic therapies, identified through MEDLINE, EMBASE, and Cochrane library (CENTRAL) up to March 31, 2025 (PROSPERO; CRD420251007431). The primary outcome was the composite of time to cardiovascular death or heart failure (HF) hospitalization. One-stage approach using stratified random-effect Cox regression model was used to re-estimate the treatment-effects for heart failure events. The relative effect of combination therapies was estimated using established methods for making indirect comparisons by an additive network meta-analysis model.

**Findings:**

Eight trials involving 25,440 patients with HFpEF were analyzed. The mean age of participants ranged between 61.7 and 72 years, 11,355 (45%) were women, 10,242 (40%) with type 2 diabetes, and 4841 (19%) with obesity. In these patients, the combination of MRA, SGLT2i, and GLP-1 RA was the most effective for all clinical HF event (P-score 1.00). Compared with placebo, this combination reduced by 58% (HR 0.42 [0.31–0.56]) the risk of combined endpoint of cardiovascular death or HF hospitalization. The corresponding estimated absolute risk reduction (ARR) over a median follow-up of 2.5 years was 6.9% (95% CI, 4.6%–8.7%]), with a number need to treat (NNT) of 14 (95% CI 12–22). There was also a risk reduction of 73% (HR 0.27 [0.18–0.42]; ARR 6.2% [4.2%–7.5%]; NNT 16 [13–24]) for HF hospitalization, and 43% (HR 0.57 [0.44–0.72]; ARR 3.0% [1.9%–4.0%]; NNT 34 [26–52]) for cardiovascular death. Among the double combinations, MRA and GLP-1 RA strategies were the most effective in the reduction in the risk of HF hospitalization (P-score 0.77) and cardiovascular death (P-score 0.75), while SGLT2 and GLP-1 RA strategies were the most effective in the reduction in the risk of the composite endpoint (P-score 0.77).

**Interpretation:**

In patients with HF and LVEF > 40% for whom few treatment options are available, adjunctive combination of MRA, SGLT2i, and GLP-1 RA to standard care has the potential to confer benefit in heart failure events (moderate quality of evidence). Furthermore, large-scale randomized combination therapies with extended follow-up are needed to confirm these findings, and to explore potential benefits in subgroups, optimized protocols, and inform clinical guidelines.

**Funding:**

None.

**Research in context**

**Evidence before this study**

Sodium-glucose cotransporter 2 inhibitors (SGLT2i), mineralocorticoid receptor antagonists (MRA), and glucagon-like peptide 1 receptor agonists (GLP-1 RA) have demonstrated significant benefits in patients with heart failure with mildly reduced or preserved ejection fraction (HFpEF), beyond the reduction of classical risk factors. We searched PubMed, EMBASE and the Cochrane Central Register of Controlled Trials (CENTRAL) for reports published before March 31, 2025 using the search terms “glucagon-like peptide 1 receptor agonists (GLP-1 RAs)”, “sodium glucose cotransporter 2 inhibitors (SGLT2i), “steroidal or non-steroidal mineralocorticoid receptor antagonists”, and “mineralocorticoid receptor antagonists (MRAs)”, “randomized controlled trials”, “death”, “heart failure (HF)”, “hospitalization for heart failure”, and “death form cardiovascular causes”. However, no trial has specifically evaluated their comparative effectiveness or the potential benefit of their combination.

**Added value of this study**

To the best of our knowledge, this systematic review is the first to investigate the impact of the relative effect of adjunctive combination to standard care of MRA, SGLT2i, and GLP-1 RA versus placebo on heart failure events in eight large-scale trials involving 25,440 patients with heart failure with HF and LVEF > 40%. Our findings suggest that combination of MRA, SGLT2i, and GLP-1 RA were the most effective for all clinical HF event (P-score 1.00), followed by the double combinations of MRA and GLP-1 RA strategies for HF hospitalization (P-score 0.77) and cardiovascular death (P-score 0.75), and SGLT2 and GLP-1 RA strategies for the composite endpoint (P-score 0.77). Compared with placebo, the combination of MRA, SGLT2i, and GLP-1 RA reduced by 58% (ARR 6.9%; NNT 14) the risk of combined endpoint of HF hospitalization or cardiovascular death, 73% (ARR 6.2%; NNT 16) the risk for HF hospitalization, and 43% (ARR 3.0%; NNT 34) the risk for cardiovascular death.

**Implications of all the available evidence**

Given the high residual risk of heart failure events in patients with HFpEF and the lack of head-to-head comparison trials, our results provide a robust basis for considering combination therapy as a strategy to further improve outcomes in this population. This evidence may inform future clinical guidelines and support the design of dedicated combination therapy trials.

## Introduction

Heart failure with mildly reduced or preserved ejection fraction (thereafter referred as HFpEF), the most common type of heart failure (HF) is associated with a high risk of hospitalization and cardiovascular (CV) death, especially in participants with overweight, obesity or type 2 diabetes.^1^ Despite this, therapeutic options for HFpEF remain limited compared to heart failure with reduced ejection fraction (HFrEF), in whom the use of combination therapies is widely supported by class 1 guidelines.

Evidences from meta-analyses showed that mineralocorticoid receptor antagonists (MRAs),^2^ sodium glucose cotransporter 2 inhibitors (SGLT2i),^3^ and glucagon-like peptide 1 receptor agonists (GLP-1 RA)^4^ have demonstrated efficacy in reducing heart failure events in patients with HFpEF. Results from a recent cross-trial synthesis detailed the potential gains obtained with combination therapies across the left ventricular ejection fraction (LVEF) spectrum.^5^ Similarly, the findings from aggregated network meta-analysis showed that, in patients with HF and LVEF > 40%, the quadruple combination of ARNI, beta-blockers (BB), MRA, and SGLT2i provides the largest reduction in the risk CV death and HF hospitalization.^6^

However, these meta-analyses did not included findings from post-hoc pooled, participant-level analyses of four placebo-RCTs including SELECT,^7^ FLOW,^8^ STEP-HFpEF,^9^ and STEP-HFpEF DM^10^ trials involving 3743 participants with history of HFpEF, most of whom were patients with overweight or obesity.^4^ These findings support the use of once-weekly semaglutide as an efficacious and safe therapy to reduce the risk of clinical heart failure events. Incorporating semaglutide trials will help to explore potential benefits of the additive effect of drug therapies, optimized protocols, and inform clinical guidelines in patient with HFpEF. In addition, the availability of the new findings of semaglutide in this population gives the opportunity to update the previous meta-analyses.

To address this gap, we conducted the present network meta-analysis using reconstructed individual patient data to identify the best therapeutic strategy in patients with HFpEF.

## Methods

### Search strategy and selection criteria

We conducted a systematic review and meta-analysis in accordance with the Preferred Reporting Items for Systematic Reviews and Meta-Analysis (PRISMA) guidelines.^11^ We searched MEDLINE (via PubMed), CENTRAL and EMBASE databases through March 31, 2025. We included published randomized controlled trials (RCTs) that evaluated the efficacy of cardiometabolic therapies on heart failure events compared with placebo in patients with HF and LVEF > 40%. To minimize the risk of small bias, and to ensure sufficient statistical power and stability in time-to-event outcomes rate estimation, especially for rare event such as cardiovascular death, we only included RCTs with over 1000 patient-years of follow-up in each randomized group. RCTs in which the Kaplan–Meier plot did not report the number of at-risk participants, and non-randomized trials were excluded. The study protocol was registered in PROSPERO (CRD420251007431).

### Ethics

Ethics committee review and/or approval was not required for this study, as it was a systematic review and network meta-analysis based exclusively on reconstructed patient-level data from previously published Kaplan–Meier curves of literature.

### Data extraction, risk of bias assessment, and certainty of evidence

**Fig. 1:**
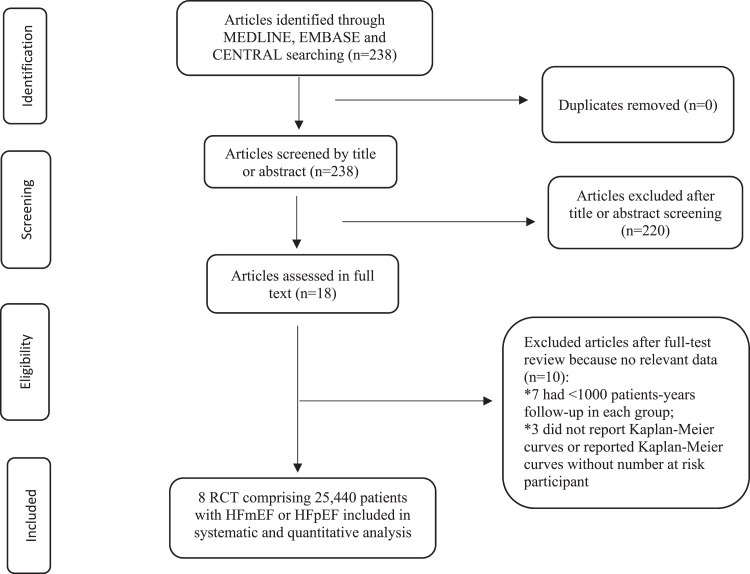
**PRISMA flowchart of 8 trials selected for meta-analysis of cardiometabolic drugs in patients with HFmEF and HFpEF.** RCT: randomized controlled trial; HFmEF: heart failure with mildly reduced ejection fraction and HFpEF: heart failure with preserved ejection fraction.

Data extraction and assessment of trial bias were performed independently by 2 reviewers (AD and MCB), with disagreements resolved by discussion until a consensus was reached. For each trial meeting the eligibility criteria, we extracted data on patients' characteristics (sex, age, BMI, type of diabetes, and obesity or overweight), trial follow-up, sample size, and cardiometabolic therapies. We extracted the Kaplan–Meier curves from the pooled individual trials of semaglutide (figure 1 in the manuscript),^4^ MRAs (figure 1 in the manuscript),^2^ EMPEROR-PRESERVED (figures 1 and 3 in the manuscript, and figure S3 from appendix),^12^ and DELIVER (figure 1 in the manuscript).^13^ Thereafter, we reconstructed the IPD by scanning the published Kaplan–Meier cumulative incidence curves using the WebPlotDigitizer software,^14^ then applying the reconstruction algorithm of Guyot and Colleagues, which uses the magnitudes and locations of steps in the Kaplan–Meier curves, together with the numbers of patients at-risk, to infer the number of events and censorings occurring within each time interval.^15^, ^16^, ^17^ The quality of evidence for each outcome and comparison in the network meta-analysis was assessed using the Confidence in Network Meta-analysis (CINeMA) framework.^18^^,^^19^ In the CINeMA tool six domains that affect the level of confidence in the network meta-analysis were considered: reporting bias, indirectness, imprecision, heterogeneity, incoherence, and within-study bias which was assessed using the Cochrane Collaboration’s revised Risk of Bias Tool (Rob2),^20^ with more details are provided in Appendix (pages 6–8). Briefly, for the within-study risk of bias, we considered the randomization sequence generation and allocation concealment (selection bias), the blinding of participant and personal (performance bias), the blinding outcome (detection bias), incomplete outcome data (attrition bias), and selective reporting (reporting bias). For the reporting bias domain, we referred to bias that can occur due to publication bias (suppression of statistically non-significant or negative finding). As there were fewer than 10 studies included in our analysis, we did not assess this risk of bias, then we assumed that the reporting bias was undetected. The indirectness was referred to the relevance to the following research question: Do the treatment benefits combination of SGLT2 inhibitors, steroid and non-steroid MRAs, or GLP-1 RA (interventions) in terms of the reduction in the risk of composite of HF hospitalization or cardiovascular death (outcome) extend to patients with HFpEF (population)? All included study have a low risk of indirectness leading to “no concerns” assignment. The imprecision was assessed by the 95% confidence intervals which may include values that could lead different clinical conclusion. To judge imprecision, we defined clinically important size effect of 1 (HR = 1), with HR < 1 and HR > 1 was considered as clinically important. Then, we assumed that imprecision corresponds to statistically non-significant associations whose confidence interval includes 1. Heterogeneity was referred to the variation in the treatment effects between studies, using the variance of the underlying treatment effects (ι^2^) to estimate the prediction interval. In our study, heterogeneity was major concerns if the prediction interval includes values that lead to a different conclusion than an assessment based on the confidence interval. Incoherence is the statistical manifestation of intransitivity, e.g., disagreement between direct and indirect comparisons. Finally, the overall CINeMA judgement across the 6 domains was summarized using the four levels: “very low (red color)”, “low (yellow color)”, “moderate (blue color)”, and “high (green color)”. Starting at high confidence and drop the level of confidence by 1 step for each domain with some concerns, and by 2 levels for each domain with major concerns. Because imprecision, heterogeneity and incoherence are interconnected, we downgraded by 2 levels in case of some concerns for two of them and major concerns for one of them.

### Statistics

The primary outcome was the composite of time to CV death or HF hospitalization event (unplanned hospitalization or urgent visit for HF as defined within each study). Secondary outcomes were time to first HF hospitalization and time to CV death, including death from stroke, coronary heart disease, myocardial infarction, or sudden cardiac death. Hazard ratios (HR) for primary and secondary outcomes were re-estimated using the one-stage approach using the stratified mixed Cox regression models with trials random-effects to account for difference in the study design and the participant baseline risk.^21^ Thereafter, we created a connected network of the different treatment combination, and then we evaluated comparison between the different treatment combinations, as well as between the individual treatment component in an additive network meta-analysis.^22^ The relative effect of treatment combination was estimated using established methods for making indirect comparisons.^23^^,^^24^ The main combination treatment effect is estimated from the multiplicative product of the treatment effects from each derivative trial (i.e., treatment effects additive on the log [hazard ratio] scale). The 95% CIs were estimated as the square root of the sum of squared standard errors of the logarithmic HRs from each of the component trials. The corresponding absolute risk reduction (ARR) and number need to treat (NNT) during a median follow-up of 2.5 years were also estimated based on the relative treatment effect applied to event rate in patients receiving placebo. To choose the preferred treatment combination, the P-score which ranging from 0 (worse treatment combination) to 1 (best treatment combination) was computed for each treatment, then treatment with a higher P-score was selected as the better than the competing each treatment. All analyses were performed using the *R ‘netmeta’* and *‘coxme’* packages. A P value < 0.05 was considered statistically significant for pooled estimates.

### Sensitivity analyses

According to the reviewers’ suggestions, we performed sensitivity analyses by excluding the comparison of MRA + SGLT2i vs GLP-1 RA therapies, which was responsible to the significant heterogeneity and inconsistency for the primary composite endpoint and hospitalizations for heart failure.

### Role of the funding source

There were no funding sources for this study.

## Results

Of 238 records screened, 18 were fully reviewed and eight RCTs met the inclusion criteria,^7^, ^8^, ^9^, ^10^^,^^12^^,^^13^^,^^25^^,^^26^ involving 25,440 patients with HFpEF (Fig. 1). Study and participant characteristics are presented in Table 1. The mean age of participants ranged between 61.7–72 years, 11,355 (45%) were women, 10,242 (40%) were patients with type 2 diabetes, 4841 (19%) were persons with obesity, 2811 (11%) had atrial fibrillation, 6193 (24%) had NYHA functional class III/IV, and 910 (4%) were Black or African American. The mean BMI ranged between 29 and 37 kg/m^2^, the mean LVEF between 52.5%–57%, and the mean NT-proBNP between 450.8 to 1040.5 pg/ml. Of the eight included trials, four (n = 3743) evaluated GLP-1 RA (semaglutide), two (n = 12,251) evaluated SGLT2i (dapagliflozin and empagliflozin), and two (n = 9446) evaluated steroid and non-steroid MRA (spironolactone and finerenone). During a median follow-up of 2.5 years (SD 0.96), 4229 patients had a composite of HF hospitalization or CV death; 3934 experienced HF hospitalization, and 1855 died from CV cause.

**Table 1:** Trials of cardiometabolic therapies meeting inclusion criteria.

	GLP-1 RA trials (four trials; n = 3743)	SGLT2 inhibitors trials (two trials; n = 12,251)	Steroid and non-steroid MRA trials (two trials; n = 9446)
Trials	SELECT, FLOW, STEP-HFpEF, and STEP-HFpEF DM	DELIVER and EMPEROR-PRESERVED	TOPCAT and FINEARTS-HF
Investigational drug	Semaglutide	Dapagliflozin and Empagliflozin	Spironolactone and Finerenone
Median follow-up time	2.3 years	2.5 years	3.0 years
Primary outcome as defined from a pooled meta-analysis	Composite of time to cardiovascular death or first worseening heart failure event (defined as hospitalization or urgent visit due to heart failure)	Time from randomization to the occurrence of the composite of cardiovascular death or hospitalization for heart failure	Composite of time to first hospitalization for heart failure or cardiovascular death
Mean age, years	64 (NR)	71.8 (9.6)	70 (9)
Sex, women	1425 (38%)	5423 (44%)	4507 (48%)
Black or African American	103 (2.8%)	417 (3.4%)	390 (4.1%)
Mean BMI, kg/m^2^	34.1 (NR)	29.5 (NR)	31.0 (6.6)
Mean LVEF %	57 (NR)	54.3% (8.8)	55% (8)
Median NT-proBNP, pg/mL	476.6 (NR)	992.5 (561.0–1741.0)	1013.5 (449.6–1929.8)
NYHA functional class III-IV	571 (15%)	2650 (22%)	2990 (32%)
Type 2 diabetes	941 (15%)	5546 (45%)	3572 (38%)
Atrial fibrillation	940 (15%)	3057 (25%)	4487 (48%)
Concomitant HF medical therapy			
ACE inhibitors or ARB	3108 (83%)	9664 (79%)	7146 (76%)
ARNI		435 (4%)	513 (9%)
B blocker	3037 (81%)	10,344 (84%)	7768 (82%)
MRA	836 (22%)	4511 (37%)	9446 (100%)
Diuretic	2273 (61%)	NR	8747 (93%)
Loop diuretic	1353 (36%)	4807 (39%)	NR
Thiazides	429 (11%)	NR	NR
SGLT2 inhibitors	258 (7%)	12,251 (100%)	817 (14%)
GLP-1 RA	3743 (100%)	NR	NR

MRA: steroid and non-steroid mineralocorticoid receptor antagonists; SGLT2i: sodium glucose cotransporter 2 inhibitors; GLP-1 RA: glucagon-like peptide 1 receptor agonists; HF: Heart failure; NR: not reported; ARNI: angiotensin-receptor neprilysin inhibitor; BMI: body mass index; LVEF: left ventricular ejection fraction; **DELIVER:** Dapagliflozin Evaluation to Improve the Lives of Patients with Preserved Ejection Fraction Heart Failure; **EMPEROR-PRESERVED**: Empagliflozin Outcome Trial in Patients with Chronic Heart Failure with Preserved Ejection Fraction; **TOPCAT**: Treatment of Preserved Cardiac Function Heart Failure with an Aldosterone Antagonist; **FINEARTS-HF**: Finerenone Trial to Investigate Efficacy and Safety Superior to Placebo in Patients with Heart Failure; **SELECT**: Semaglutide Effects on Cardiovascular Outcomes in People with Overweight or Obesity; **FLOW**: Evaluate Renal Function with Semaglutide Once Weekly; **STEP-HFpEF**: Once Weekly on Function and Symptoms in Subjects with Obesity-related Heart Failure with Preserved Ejection Fraction; and **STEP-HFpEF DM**: Semaglutide Treatment Effect in People with Obesity and Heart Failure with Preserved Ejection Fraction and Diabetes Mellitus.

**Fig. 2:**
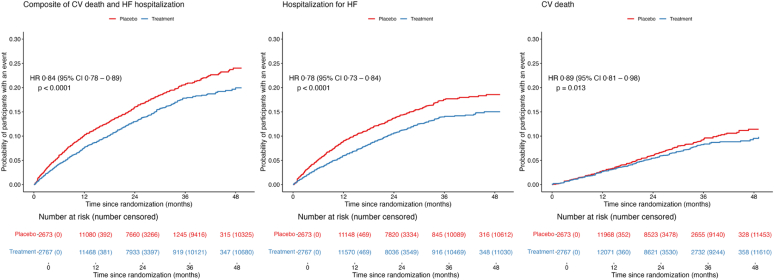
**Time to cardiovascular death or hospitalization for heart failure events (composite endpoint) (left), hospitalization for heart failure (medium), and cardiovascular death (right).** HF: Heart failure; CV: cardiovascular; HR: hazard ratio; CI: confidence interval. The cumulative incidence rate was calculated using the Aalen-Johansen method.

**Fig. 3:**
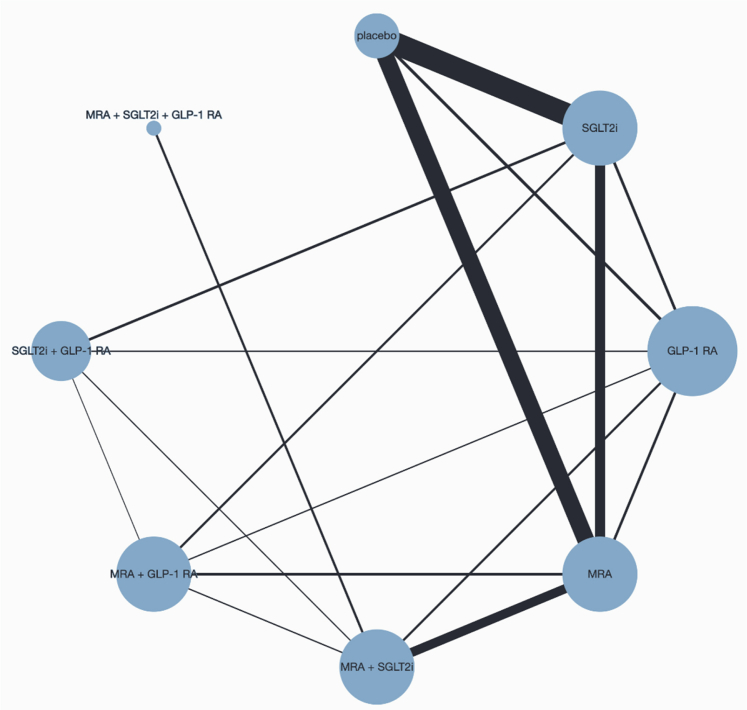
**Network graph of eligible heart failure with mildly reduced or preserved ejection fraction treatment comparison for efficacy.** Line width is proportional to the inverse variance of treatment effect size comparing every pair of treatment. The size of circle is proportional to the number of comparisons. MRA: steroid and non-steroid mineralocorticoid receptor antagonists; SGLT2i: sodium glucose cotransporter 2 inhibitors; GLP-1 RA: glucagon-like peptide 1 receptor agonists.

**Fig. 4:**
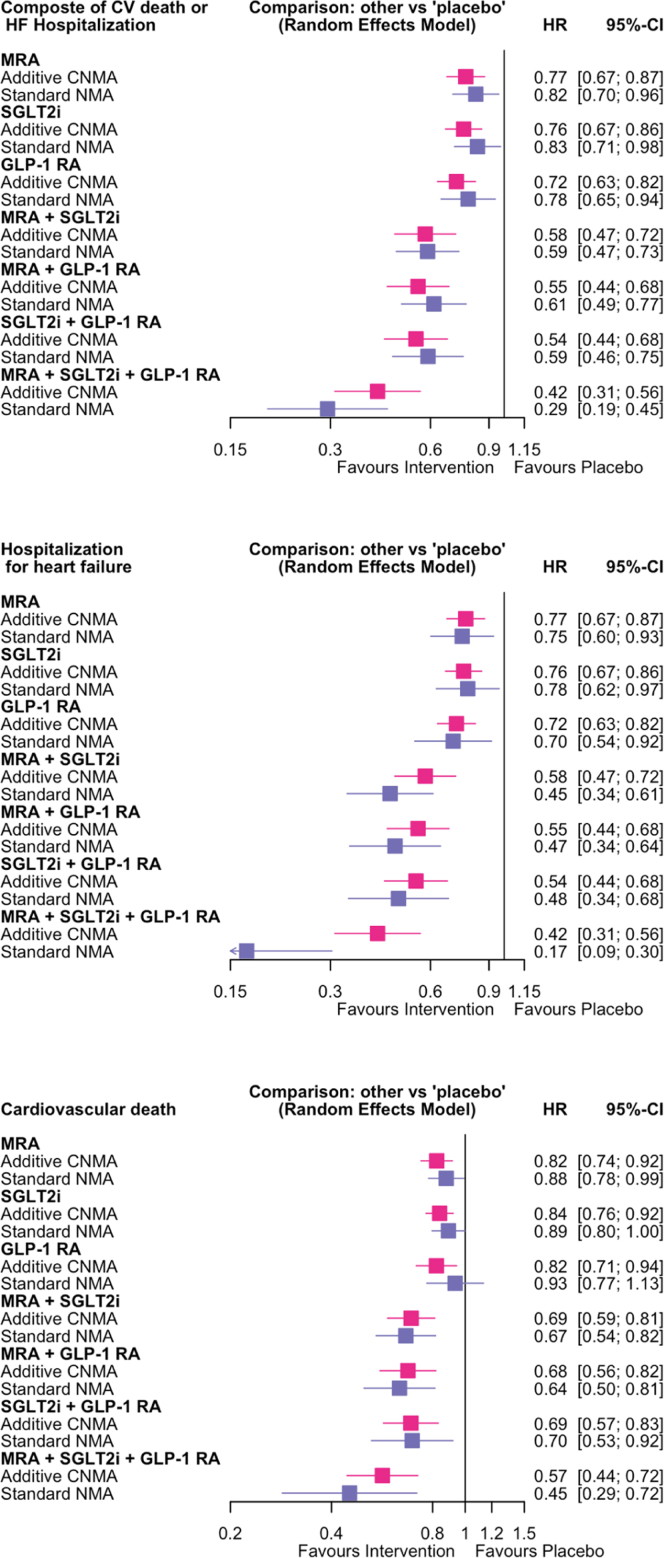
**Combination of treatment-effect on composite of CV death or hospitalization for HF (composite primary outcome) (top), HF hospitalization (medium), and CV death (bottom).** MRA: steroid and non-steroid mineralocorticoid receptor antagonists; SGLT2i: sodium glucose cotransporter 2 inhibitors; GLP-1 RA: glucagon-like peptide 1 receptor agonists; HF: Heart failure; CV: cardiovascular; HR: hazard ratio; CI: confidence interval; CNMA: combined network meta-analysis; NMA: network meta-analysis.

Overall, compared with placebo, cardiometabolic therapies reduced the risk of the composite endpoint of CV death or HF hospitalization (HR 0.84 [95% CI 0.78–0.89]; P < 0.0001; Fig. 2A). The corresponding absolute risk reduction (ARR) over a median follow-up of 2.5 years was 3.1% (95% CI 2.1%–4.2%), with a number need to treat (NNT) of 33 (95% CI 24–48). Fig. 3 shows the network for efficacy captured by the cardiometabolic treatments involving 28 pairwise comparisons from four pairs of treatment combinations (three dual therapies and one triple therapies). In the additive network meta-analysis (NMA), the combination of MRA, SGLT2i, and GLP-1 RA was the most effective for reducing the risk of the primary composite outcome (P-score 1.00; Appendix Table S4), followed by SGLT2i and GLP-1 RA (P-score 0.77), MRA and GLP-1 RA (P-score 0.74), and MRA and SGLT2i (P-score 0.63). Compared with placebo, the combination of MRA, SGLT2i, and GLP-1 RA was associated with a 58% reduction in the risk of the primary endpoint (HR 0.42 [0.31–0.56]; ARR 6.9% [4.6%–8.7%]; NNT 14 [12–22]; Fig. 4A). Consistently, the standard NMA estimated a 71% risk reduction (HR 0.29 [0.19–0.45]), suggesting a potentially larger effect when considering treatment combinations as unique interventions. The corresponding risk reductions were 50% (HR 0.50 [0.35–0.71]), 50% (HR 0.50 [0.33–0.76]), 52% (HR 0.48 [0.32–0.72]), 62% (HR 0.38 [0.25–0.57]), 64% (HR 0.36 [0.24–0.53]), and 65% (HR 0.35 [0.24–0.53]) compared with SGLT2i and GLP-1 RA, MRA and SGLT2i, MRA and GLP-1 RA, GLP-1 RA, MRA, and SGLT2i strategies respectively (Appendix Table S1). No difference was observed between the three pair of double combinations (SGLT2i and GLP-1 RA, MRA and SGLT2i, MRA and GLP-1 RA). Additionally, there was no difference between SGLT2i, GLP-1 RA, and MRA therapies (Appendix Table S1).

Cardiometabolic therapies also significantly lowered the risk of HF hospitalization (1237 [9.7%] vs 1674 [13.2%]; HR 0.78 [0.73–0.84]; P < 0.0001; ARR 3.5% [2.6%–4.3%]; NNT 28 [23–39]; Fig. 2B). In the additive NMA, the combination of MRA, SGLT2i, and GLP-1 RA was the most effective in reducing the risk of HF hospitalization (P-score 1.00; Appendix Table S4), followed by MRA and GLP-1 RA (P-score 0.77), SGLT2i and GLP-1 RA (P-score 0.74), and MRA and SGLT2i (P-score 0.64). Compared with placebo, in the additive NMA, the triple combination of MRA, SGLT2i, and GLP-1 RA was associated with a 73% reduction in the risk of HF hospitalization (0.27 [0.18–0.42]; ARR 6.2% [4.2%–7.5%]; NNT 16 [13–24]; Fig. 4B). Consistently, the standard NMA estimated an 83% risk reduction (HR 0.17 [0.09–0.30]). When compared with other treatment strategies, the triple combination was associated with risk reductions ranging from 63% to 78% (Appendix Table S2). No significant differences were observed between the three double combination strategies (SGLT2i and GLP-1 RA, MRA and SGLT2i, MRA and GLP-1 RA), nor between individual therapies (SGLT2i, GLP-1 RA, and MRA) (Appendix Table S2).

As for primary endpoint and HF hospitalization, cardiometabolic therapies significantly lowered the risk of CV death (878 [6.7%] vs 977 [7.7%]; HR 0.89 [0.81–0.98]; P = 0.0174; ARR 0.8% [0.2%–1.4%]; NNT 120 [69–658]; Fig. 2C). In the additive NMA, the triple combination of MRA, SGLT2i, and GLP-1 RA was the most effective in reducing the risk of CV death (P-score 0.99; Appendix Table S4), followed by MRA and GLP-1 RA (P-score 0.75), SGLT2i and GLP-1 RA (P-score 0.69), and MRA and SGLT2i (P-score 0.69). Compared with placebo, the triple combination of MRA, SGLT2i, and GLP-1 RA was associated with a 43% reduction in the risk of CV death (0.57 [0.44–0.72]; ARR 3.0% [1.9%–4.0%]; NNT 34 [26–52]; Fig. 4C). Consistently, the standard NMA estimated a 55% risk reduction (HR 0.45 [0.29–0.72]). When compared with individual therapies, the corresponding risk reductions were 48%–52% (Appendix Table S3). Unlike for the primary endpoint and HF hospitalization, no significant difference was observed between the triple combination and the double combination strategies (Appendix Table S3).

Because of differences in adverse events ascertainment and reporting in both trials, we did not perform directedly safety outcomes comparison or meta-analyzed. Nevertheless, any serious adverse events occurred numerically less frequently in the treatment groups compared with in the placebo group, while those adverse events leading to the treatment discontinuation were distributed equally between the two treatment arms except in TOPCAT trial (16.4% vs 8.2%; Appendix Table S5).^26^

Overall, except for the composite outcome and HF hospitalization in DELIVER and EMPEROR-PRESERVED trials,^12^^,^^13^ there was a violation of proportional hazard assumption (Appendix Fig. S1). The quality of evidence for benefit of outcomes from the NMA was classified as high to moderate for primary endpoint by CINeMA criteria (Appendix Fig. S2). The corresponding quality of evidence was classified as lower for HF hospitalization due to high heterogeneity and imprecision (Appendix Fig. S3), whereas the quality of evidence for CV death was rated as high to low due to the imprecision (Appendix Fig. S4). As suggested by the reviewers, we performed sensitivity analyses by excluding the comparison of MRA + SGLT2i vs GLP-1 RA, which was responsible to the significant heterogeneity and inconsistency for the primary composite endpoint and hospitalizations for heart failure. Overall, the results of the sensitivity analyses were similar to those of the main analyses, with a significant reduction in the extent of heterogeneity and inconsistency (the quality of evidence changed from “very low” to “moderate”), suggesting the robustness of our conclusions (Appendix Figs. S8–S11 and Appendix Tables S6–S8).

## Discussion

This network meta-analysis using reconstructed individual patient data of eight large trials involving 25,440 adult patients with HFpEF showed that the triple combination of MRA, SGLT2i, and GLP-1 RA added to standard care was the most effective strategy for reducing the composite outcome of cardiovascular death or HF hospitalization. Additionally, this combination lowered significantly the risk of both HF hospitalization and CV death.

Importantly, double combination therapies demonstrated similar effectiveness across heart failure events suggesting that the choice of combination may be guided by tolerance, clinical presentation and patient preference. Despite a lower proportion of CV death in this population, which are reflected in the observed NNT values, the key message is that, the adjunction to standard care of the triple combination of MRA, SGLT2i, and GLP-1 RA was associated with a 43% relative risk reduction in CV death, corresponding to an NNT of 34 to prevent one CV death.

HFpEF is a complex disease characterized by multiple phenotypes and pathophysiological mechanisms, accordingly its management requires therapies targeting different pathways to achieve optimal outcomes. As no formal interaction between available therapies was observed, it is plausible that their benefits are complementary and additive. For example, the clinical benefits of SGLT2i have been shown to be consistent, irrespective of background use of ARNI or MRA.^3^^,^^12^ Similarly, the relative effect of semaglutide on the composite outcome of CV death and HF hospitalization was be found consistent across subgroup of participants receiving (vs not receiving) MRA and SGLT2i at baseline.^4^ Beyond HFpEF, the question of optimal cardiometabolic therapies combinations was previously addressed in population with type 2 diabetes. Neuen et al.^27^ analyzed data from two trials involving SGLT2i (CANVAS and CREDENCE), two trials involving non-steroidal MRAs (FIDELIO-DKD and FIGARO-DKD), and eight trials involving GLP-1 AR to compare the effects of combination therapy with placebo on CV, kidney and mortality outcomes in patients with type 2 diabetes and moderately elevated albuminuria. Findings suggested that combining SGLT2i, GLP-1 RA and non-steroidal MRAs may offer significant benefits in for CV and kidney health, as well as overall survival.^27^

Before this study, there was limited evidence regarding the benefits of combining of steroidal and non-steroidal MRA, SGLT2i, and GLP-1 RAs in patients with HFpEF.^28^^,^^29^ A previous cross-trial synthesis reported potential benefits of combining MRA (spironolactone), ARNIs, and SGLT2is among patients with LVEF between 40% and 65%. However, CV death was reduced only in the LVEF between 40% and 55% subgroups.^5^ Additionally, an additive network meta-analysis reported a minimal incremental benefits when BB were added to the combination of MRA (spironolactone), ARNI, and SGLT2i.^6^ The recent completion of the GLP-1 RA trials using semaglutide^4^ and the FINEARTS-HF trial evaluating finerenone^25^ has expanded the therapeutic landscape for patients with heart failure and LVEF up to 40%. To date, the pooled data of four semaglutide trials now represents the largest dataset assessing GLP-1 RA effects in HFpEF, providing more precise estimates of treatment benefits. Findings from the FINEARTS-HF trial confirmed the efficacy of non-steroidal mineralocorticoid receptor blockade in this population.^25^ Unlike previous meta-analyses, we have included these new important findings, which is crucial for both clinical decisions making and practice guidelines.

The present study has several strengths. This is the first study to provide information about the effect of a combination of steroidal and non-steroidal MRAs, SGLT2is, and GLP-1 RAs in addition to standard care on heart failure events in patients with HFpEF. Second, we used reconstructed IPD with standardized treatment-effects estimation to account for patients baseline risk differences between patients due to variations in heart failure phenotyping across trials. The extracted data exactly matches that reported in the original publications, suggesting the robustness of our results (Appendix Figs. S5–S7). Third, the protocol for this study was developed a priori and registered in PROSPERO, and included a prospective definition of our research question, trial inclusion criteria (which were extended to include MRA data), primary and secondary endpoints, and a rigorous methodology. Four, inclusion of large trials (>1000 patient-years of follow-up in each randomized group) increased the power of this study to detect rare events (e.g., CV death), and reduced the risk of publication bias due to small study effects. Five, the results of the sensitivity and main analyses were similar, suggesting the robustness of our conclusions.

However, this study has several limitations. First, heterogeneity and incoherence were observed for some comparisons. The definition of clinically important size effect (pre-specified range of clinical equivalence), differences in participant background and characteristics, or the small number of included trials, may account for this heterogeneity, and caution is therefore warranted when interpreting these effects. For example, the TOPCAT included patients enrolled from 2006 to 2012 with changes in background care since that period,^26^ where SGLT2 inhibitors are currently considered a foundational treatment for heart failure. Second, the lack of patient-level data limits the ability to explore possible sources of heterogeneity, to control for potential confounders between trials, and to allow rigorous investigation of effect-modifying variables. Third, while the reconstructed data enabled standardized time-to-event analysis, it may introduce bias in treatment-effect estimates as compared with original participant-level data due to their assumption on uniform censoring and constant event rates between time points. Additionally, a potential aggregation bias can impact the treatment-effect estimates because the inability to derive separate KM curves for different subgroups or to model the joint effects of covariate, with the extent of the bias increases with the strength of the covariate effect. However, this is an issue for all meta-analyses where a covariate adjustment could not be performed.^15^ Four, although the combination therapy including GLP-1 receptor agonists appears promising, it is important to note that most GLP-1 RA trials except for FLOW trial^26^ were conducted in populations with obesity, often using high doses. Consequently, the mean BMI in these studies was significantly higher than in the general population. There is a lack of evidence regarding the efficacy and safety of GLP-1RAs in individuals without obesity or CKD. Therefore, the generalizability of these findings to non-obese, non-CKD populations remains uncertain and must be interpreted with caution. Five, we could not perform comparison between steroidal (spironolactone)^26^ with non-steroidal (finerenone) MRAs,^25^ although this would be clinically relevant given their differences. Unlike spironolactone, finerenone is a class with different physiochemical properties that block the mineralocorticoid receptor with a non-steroidal MRA.^2^ However, even the recent patient-level meta-analysis that evaluated the efficacy and safety of MRA on heart events was unable to make a direct comparison between steroid and non-steroid MRA. An appropriate well designed and powered prospective randomized trails are needed to determine whether there is a class effect of MRAs in heart failure with mildly reduced or preserved ejection fraction. Six, only 4% of participants meta-analyzed were Black or African American, limiting the generalizability of these findings in this high heart failure risk group population. Finally, we did not perform directedly adverse events comparison or meta-analyzed because of differential data capture and exact definitions of these safety events in both trials. Additionally, the safety outcomes such as hyperkaliemia which was a safety concern with MRAs and the common decline in eGFR after initiation of an MRA and SGLT2i^2^^,^^3^ were not addressed in this analysis. Nevertheless, as reported in the original included trials,^2^, ^3^, ^4^ any serious adverse events occurred numerically less frequently in the treatment groups compared with in the placebo group (Appendix). A comprehensive patient-level meta-analysis is needed to deal with cardiometabolic therapies safety outcomes. Such safety data are essential for adapting treatment, particularly in patients with end-stage kidney disease.

In conclusion, this additive network meta-analysis of almost 25,500 patients across eight large, randomized trials provides comprehensive evidence (moderate quality of evidence) that the combination of steroid and non-steroid MRA, SGLT2i, and GLP-1 RA was the most effective strategy for lowering the risk of cardiovascular death or HF hospitalization in patients with HFpEF. This combination should be considered in addition to standard care in patients with HFpEF who do not have any contraindications. Furthermore, large-scale randomized combination therapies trials with extended follow-up are needed to confirm these findings, explore potential benefits in subgroups, optimize protocols, and inform clinical guidelines.

## Contributors

Alhassane Diallo (AD) conceived, organized, designed the study plan, analyzed the data, and drafted the first version of the manuscript, Miguel Carlos-Bolumbu (MCB) collected and supervised the data collection, and critically reviewed the manuscript, Philippe Duc (PD) interpreted the analysis and extensively revised the manuscript, and Florence Galtier (FG) conceived and organized the study plan, and interpreted and extensively revised the manuscript. All authors read and approved the final version of the manuscript. All authors had access to and verify the underlying data.

## Data sharing statement

Data and R code that support the findings of this study are available from the corresponding author upon reasonable request (djuhany@gamil.com).

## Declaration of interests

AD, MCB, PD and FG declare no competing interests.
