# Supplementary material for: Prevention of cardiovascular events in heart failure with mildly reduced or preserved ejection fraction: a comprehensive network meta-analysis of eight randomized controlled trials using reconstructed individual patient’s data
Source: eClinicalMedicine. 2025 Sep 12;88:103506. doi: 10.1016/j.eclinm.2025.103506 (PMC12572806; doi:10.1016/j.eclinm.2025.103506)

**Appendix**

**eFigure S1: Hazard proportional assumption for composite of CV death or HF hospitalization (left-top panel), HF hospitalization (right-top panel), and CV death (left-bottom panel)**

**
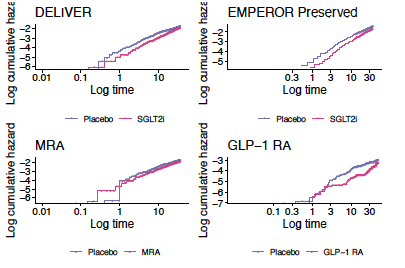
**
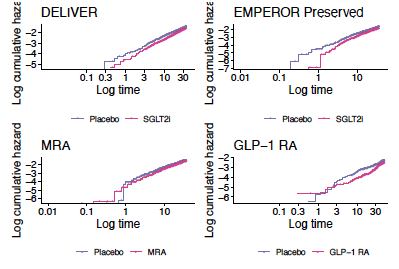


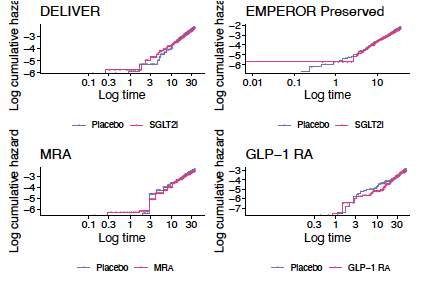


CV: cardiovascular; HF: heart failure;

**eTable S1: Ligue table from standard network meta-analysis of composite of CV death or HF hospitalization**

| **MRA + SGLT2i + GLP-1 RA**  **[P-score = 0.9999]** | |  |  |  |  |  |  |
| --- | --- | --- | --- | --- | --- | --- | --- |
| **0.50 (0.35-0.71)** | **MRA + SGLT2i [0.7376]** | |  |  |  |  |  |
| **0.50 (0.33-0.76)** | 1.00 (0.79-1.25) | **SGLT2i + GLP-1 RA [0.7303]** | |  |  |  |  |
| **0.48 (0.32-0.72)** | 0.96 (0.77-1.18) | 0.96 (0.76-1.22) | **MRA + GLP-1 RA [0.6707]** | |  |  |  |
| **0.38 (0.25-0.57)** | **0.75 (0.62-0.92)** | **0.76 (0.60-0.95)** | **0.79 (0.64-0.97)** | **GLP-1 RA [0.3575)** | |  |  |
| **0.36 (0.24-0.53)** | **0.72 (0.60-0.85)** | **0.72 (0.57-0.90)** | **0.75 (0.62-0.91)** | 0.95 (0.80-1.13) | **MRA [0.2622]** | |  |
| **0.35 (0.24-0.53)** | **0.71 (0.58-0.87)** | **0.71 (0.57-0.88)** | **0.74 (0.60-0.90)** | 0.94 (0.78-1.12) | 0.99 (0.85-1.15) | **SGLT2i [0.2384]** | |
| **0.29 (0.19-0.45)** | **0.59 (0.47-0.73)** | **0.59 (0.46-0.75)** | **0.61 (0.49-0.77)** | **0.78 (0.65-0.94)** | **0.82 (0.70-0.96)** | **0.83 (0.71-0.98)** | **Placebo (0.0035]** |

**eTable S2: Ligue table from standard network meta-analysis of HF hospitalization**

| **MRA + SGLT2i + GLP-1 RA [P-score =0.9999]** | |  |  |  |  |  |  |
| --- | --- | --- | --- | --- | --- | --- | --- |
| **0.37 (0.22- 0.62)** | **MRA + SGLT2i [0.7467]** | |  |  |  |  |  |
| **0.35 (0.19- 0.64)** | 0.94 (0.68- 1.30) | **SGLT2i + GLP-1 RA [0.7085]** | |  |  |  |  |
| **0.36 (0.20- 0.65)** | 0.97 (0.72- 1.30) | 1.02 (0.73- 1.44) | **MRA + GLP-1 RA [0.6845]** | |  |  |  |
| **0.24 (0.13- 0.43)** | **0.65 (0.49- 0.85)** | **0.68 (0.49- 0.95)** | **0.67 (0.50- 0.90)** | **GLP-1 RA [0.3550]** |  |  |  |
| **0.22 (0.13- 0.40)** | **0.61 (0.48- 0.77)** | **0.64 (0.47- 0.89)** | **0.63 (0.48- 0.83)** | 0.94 (0.74- 1.20) | **MRA [0.2788]** |  |  |
| **0.22 (0.12- 0.39)** | **0.58 (0.44- 0.77)** | **0.62 (0.45- 0.85)** | **0.60 (0.45- 0.80)** | 0.90 (0.70- 1.16) | 0.96 (0.78- 1.18) | **SGLT2i [0.2235]** |  |
| **0.17 (0.09- 0.30)** | **0.45 (0.34- 0.61)** | **0.48 (0.34- 0.68)** | **0.47 (0.34- 0.64)** | **0.70 (0.54- 0.92)** | **0.75 (0.60- 0.93)** | **0.78 (0.62- 0.97)** | **Placebo [0.0030]** |

MRA: steroid and non-steroid mineralocorticoid receptor antagonists; SGLT2i: sodium glucose cotransporter 2 inhibitors; GLP-1 RA: glucagon-like peptide 1 receptor agonists; HF: Heart failure; CV: cardiovascular; HR: hazard ratio; CI: confidence interval; value in bracket is the P-score ranking value; bold value is significant comparisons.

**eTable S3: Ligue table from standard network meta-analysis of CV death**

| **MRA + SGLT2i + GLP-1 RA [P-score = 0.9751]** | |  |  |  |  |  |  |
| --- | --- | --- | --- | --- | --- | --- | --- |
| 0.71 (0.43-1.16) | **MRA + GLP-1 RA [0.7727]** | |  |  |  |  |  |
| 0.68 (0.45-1.03) | 0.96 (0.74-1.25) | **MRA + SGLT2i [0.7165]** | |  |  |  |  |
| 0.65 (0.39-1.08) | 0.92 (0.67-1.26) | 0.96 (0.71-1.29) | **SGLT2i + GLP-1 RA [0.6595]** | |  |  |  |
| **0.52 (0.33-0.81)** | **0.73 (0.58-0.92)** | **0.76 (0.63-0.91)** | 0.79 (0.60-1.05) | **MRA [0.3373]** | |  |  |
| **0.51 (0.32-0.81)** | **0.72 (0.56-0.91)** | **0.75 (0.61-0.92)** | 0.78 (0.60-1.02) | 0.99 (0.87-1.12) | **SGLT2i [0.3016]** | |  |
| **0.48 (0.30-0.78)** | **0.68 (0.53-0.89)** | **0.71 (0.57-0.90)** | **0.74 (0.56-0.99)** | 0.94 (0.78-1.14) | 0.95 (0.79-1.16) | **GLP-1 RA [0.1957]** | |
| **0.45 (0.29-0.72)** | **0.64 (0.50-0.81)** | **0.67 (0.54-0.82)** | **0.70 (0.53-0.92)** | **0.88 (0.78-0.99)** | 0.89 (0.80-1.00) | 0.93 (0.77-1.13) | **Placebo [0.0416]** |

MRA: steroid and non-steroid mineralocorticoid receptor antagonists; SGLT2i: sodium glucose cotransporter 2 inhibitors; GLP-1 RA: glucagon-like peptide 1 receptor agonists; HF: Heart failure; CV: cardiovascular; HR: hazard ratio; CI: confidence interval; value in bracket is the P-score ranking value; bold value is significant comparisons.

**eTable S4: Ranking P-score value for clinical outcomes from additive network meta-analysis**

| **Comparison** | **P score** |
| --- | --- |
| **A composite of CV death or HF hospitalization** |  |
| MRA + SGLT2i + GLP-1 RA | 1.0000 |
| SGLT2i + GLP-1 RA | 0.7677 |
| MRA + GLP-1 RA | 0.7407 |
| MRA + SGLT2i | 0.6325 |
| GLP-1 RA | 0.3675 |
| SGLT2i | 0.2593 |
| MRA | 0.2323 |
| Placebo | 0.0000 |
| **HF hospitalization** |  |
| MRA + SGLT2i + GLP-1 RA | 1.0000 |
| MRA + GLP-1 RA | 0.7665 |
| SGLT2i + GLP-1 RA | 0.7351 |
| MRA + SGLT2i | 0.6401 |
| GLP-1 RA | 0.3599 |
| MRA | 0.2649 |
| SGLT2i | 0.2335 |
| Placebo | 0.0000 |
| **CV death** |  |
| MRA + SGLT2i + GLP-1 RA | 0.9996 |
| MRA + GLP-1 RA | 0.7477 |
| SGLT2i + GLP-1 RA | 0.6926 |
| MRA + SGLT2i | 0.6921 |
| GLP-1 RA | 0.3079 |
| MRA | 0.3074 |
| SGLT2i | 0.2523 |
| Placebo | 0.0004 |

MRA: steroid and non-steroid mineralocorticoid receptor antagonists; SGLT2i: sodium glucose cotransporter 2 inhibitors; GLP-1 RA: glucagon-like peptide 1 receptor agonists; HF: Heart failure; CV: cardiovascular; HR: hazard ratio; CI: confidence interval;

**eFigure S2: The quality of evidence by the Confidence in Network Meta-analysis (CINeMA) criteria for a composite of CV death or HF hospitalization**


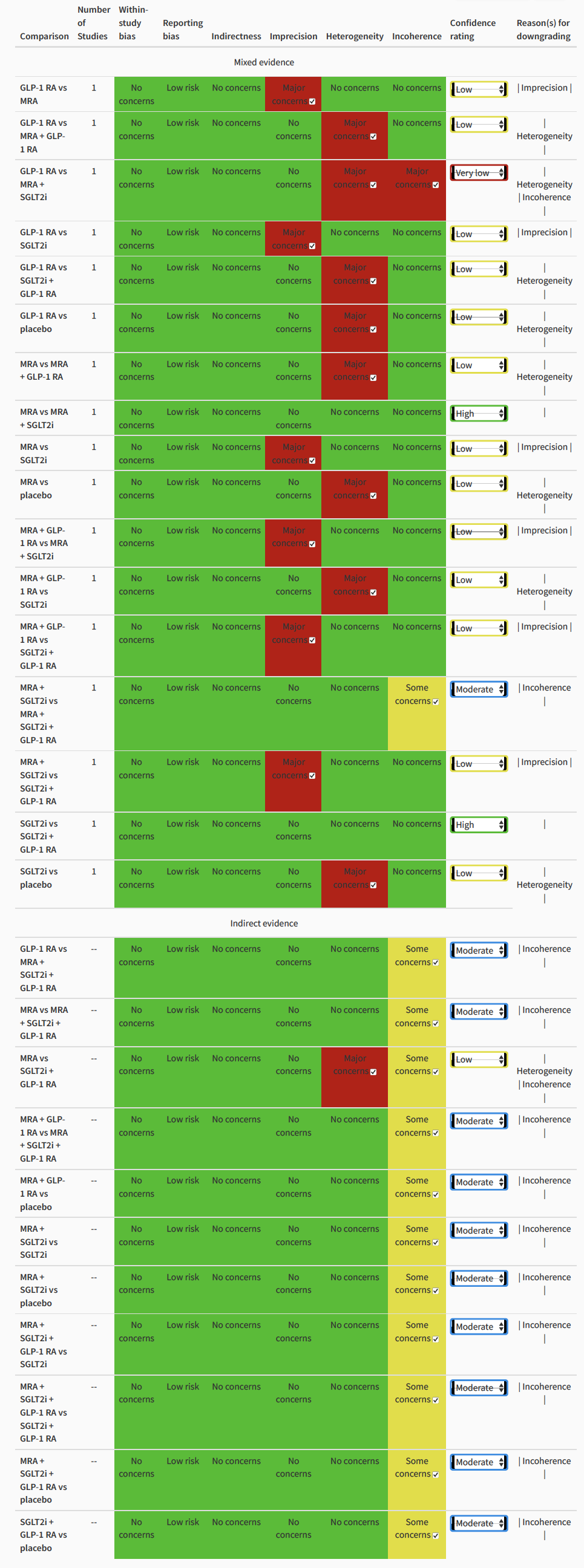


e**Figure S3: The quality of evidence by the Confidence in Network Meta-analysis (CINeMA) criteria for HF hospitalization**

**
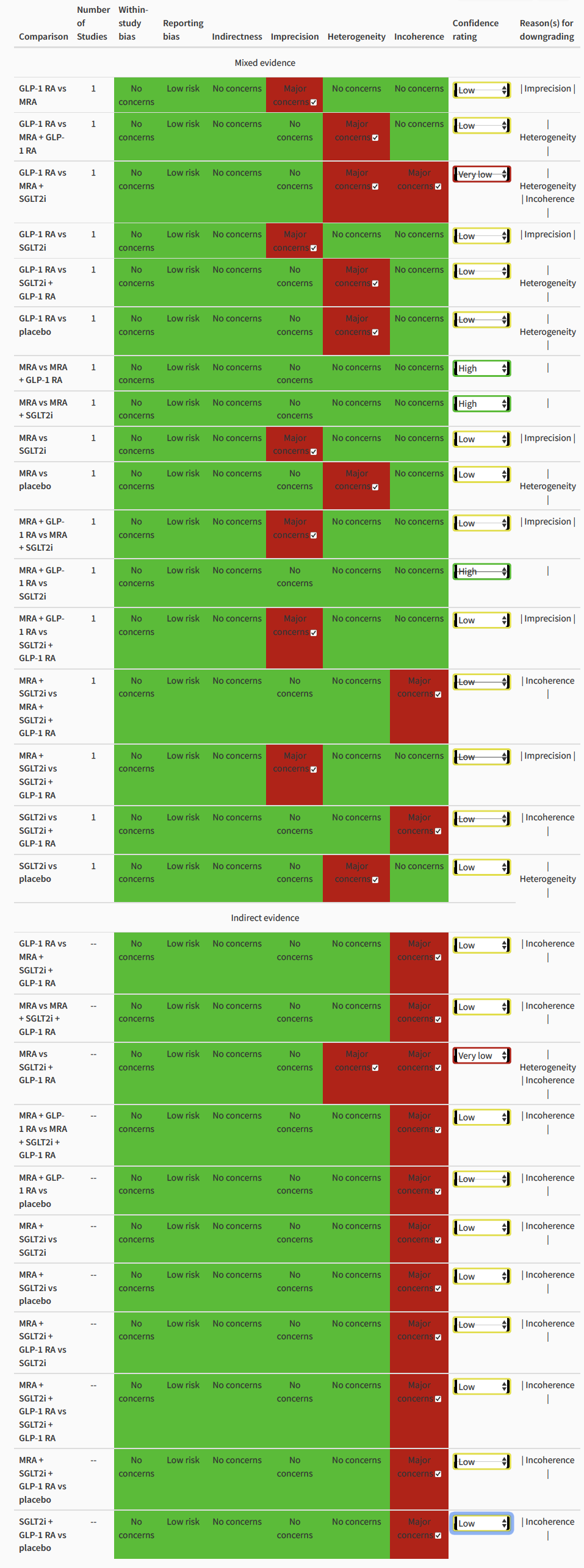
**

**eFigure S4: The quality of evidence by the Confidence in Network Meta-analysis (CINeMA) criteria for CV death**

**
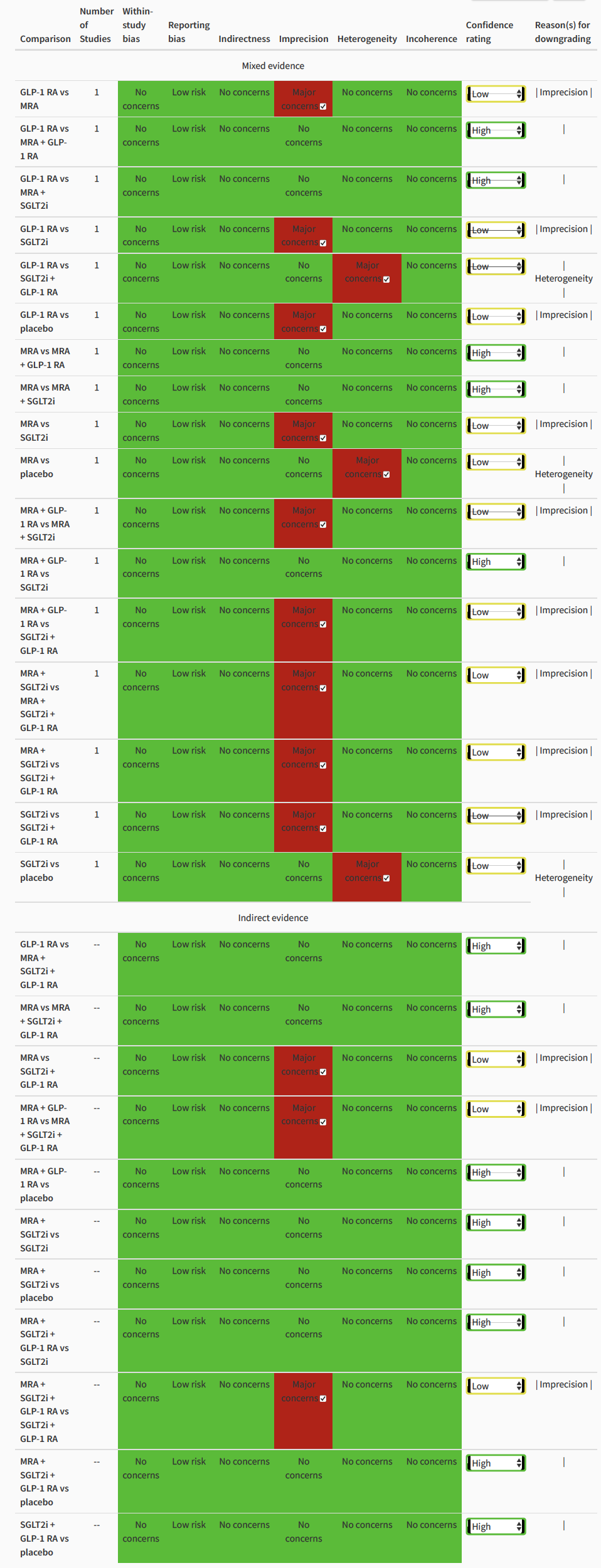
**

The quality of evidence for each outcome and comparison in the network meta-analysis was assessed using the Confidence in Network Meta-analysis (CINeMA) framework. In the CINeMA tool six domains that affect the level of confidence in the network meta-analysis were considered: reporting bias, indirectness, imprecision, heterogeneity, incoherence, and within-study bias.

1-Within-study bias:

The within-study risk of bias was assessed using the revised Cochrane Collaboration’s Risk of Bias Tool (Rob2) for RCTs. In Rob2, the following characteristics were considered: randomization sequence generation and allocation concealment (selection bias), the blinding of participant and personal (performance bias), the blinding outcome (detection bias), incomplete outcome data (attrition bias), and selective reporting (reporting bias). These answers lead to judgement of “high risk of bias”, “some concerns”, and “low risk of bias”. Then, the study-level judgment can be combined with the percentage contribution matrix to produce a bar chart, and it can be useful to assign judgment from the CINeMA tool of “no concerns (green color)”, “some concerns (yellow color)”, and “major concerns (red color)” about within-study bias according to the “low risk of bias”, “some concerns”, and “high risk of bias”. In our analysis, all included study have a low risk of bias leading to “no concerns” assignment (figures S2 – S4).

2-Reporting bias:

In our analysis, the reporting bias domain refers to bias that can occur due to publication bias (suppression of statistically non-significant or negative finding). CINeMA assume two levels of judgment for reporting bias: “suspected (red color)” and “undetected (green color)”. As there were fewer than 10 studies included in our analysis, we did not assess this risk of bias, then we assumed that the reporting bias was undetected.

3-Indirectness:

The indirectness refers to the relevance to the research question for each included study, with a clearly defined population, intervention and outcomes characteristics. The judgment and rating are like the within-study bias. In this study, the research question is: Do the treatment benefits combination of SGLT2 inhibitors, steroid and non-steroid MRAs, or GLP-1 RA (interventions) in terms of the reduction in the risk of composite of HF hospitalization or cardiovascular death (outcome) extend to patients with heart failure with mildly reduced or preserved ejection fraction (population)? All included study have a low risk of indirectness leading to “no concerns” assignment.

4-Imprecision:

The imprecision is assessed by 95% confidence intervals which may include values that could lead different clinical conclusion. To judge imprecision, users are asked to define a clinically important size of effect on the scale of the select effect measure. We defined clinically important size effect of 1 (HR=1), with HR<1 and HR>1 is considered as clinically important. Then, we assumed that imprecision corresponds to statistically non-significant associations whose confidence interval includes 1.

5-Heterogeneity:

Heterogeneity refers to the variation in the treatment effects between studies, but also, variation between direct and indirect sources of evidence (called incoherence). Heterogeneity and incoherence are related, with incoherence can be seen as a special form of heterogeneity. We measured heterogeneity using the variance of the underlying treatment effects (ι^2^) to estimate the prediction interval. As for imprecision, the CINeMA approach to heterogeneity involves comparisons results the pre-specified range of clinical equivalence. In our study, as a defined clinically important size effect was set of 1, heterogeneity is major concerns if there was a disagreement between prediction intervals and confidence interval (e.g., prediction interval includes values that lead to a different conclusion than an assessment base on the confidence interval).

6-Incoherence:

Transitiviy stipules that we can compare 2 treatments indirectly via an intermediate treatment node. Incoherence is the statistical manifestation of intransitivity, e.g., disagreement between direct and indirect comparisons. Ratio of direct and indirect treatment effects estimations (e.g., HR) with their 95% confidence interval is used inconsistency measures. According to the value of the corresponding P value of the global design-by-treatment interaction, there are “major concerns” if (P value < 0.05), “some concerns” if (P value between 0.05 and 0.10), and “no concerns” if (P value > 0.10).

7-Summarising judgement across the 6 domains:

As in GRADE approach, the overall CINeMA judgement across the 6 domains is summarized using the four levels: “very low (red color)”, “low (yellow color)”, “moderate (blue color)”, and “high (green color)”. Starting at high confidence and drop the level of confidence by 1 step for each domain with some concerns, and by 2 levels for each domain with major concerns. Because imprecision, heterogeneity and incoherence are interconnected, we downgraded by 2 levels in case of some concerns for two of them and major concerns for one of them. For example, if we have some concerns for imprecision and heterogeneity, and major concerns for incoherence, downgrading by 2 levels was applied.

**eFigure S5: reconstructed time-to the composite of CV death or HF hospitalization for individual included trials**

**
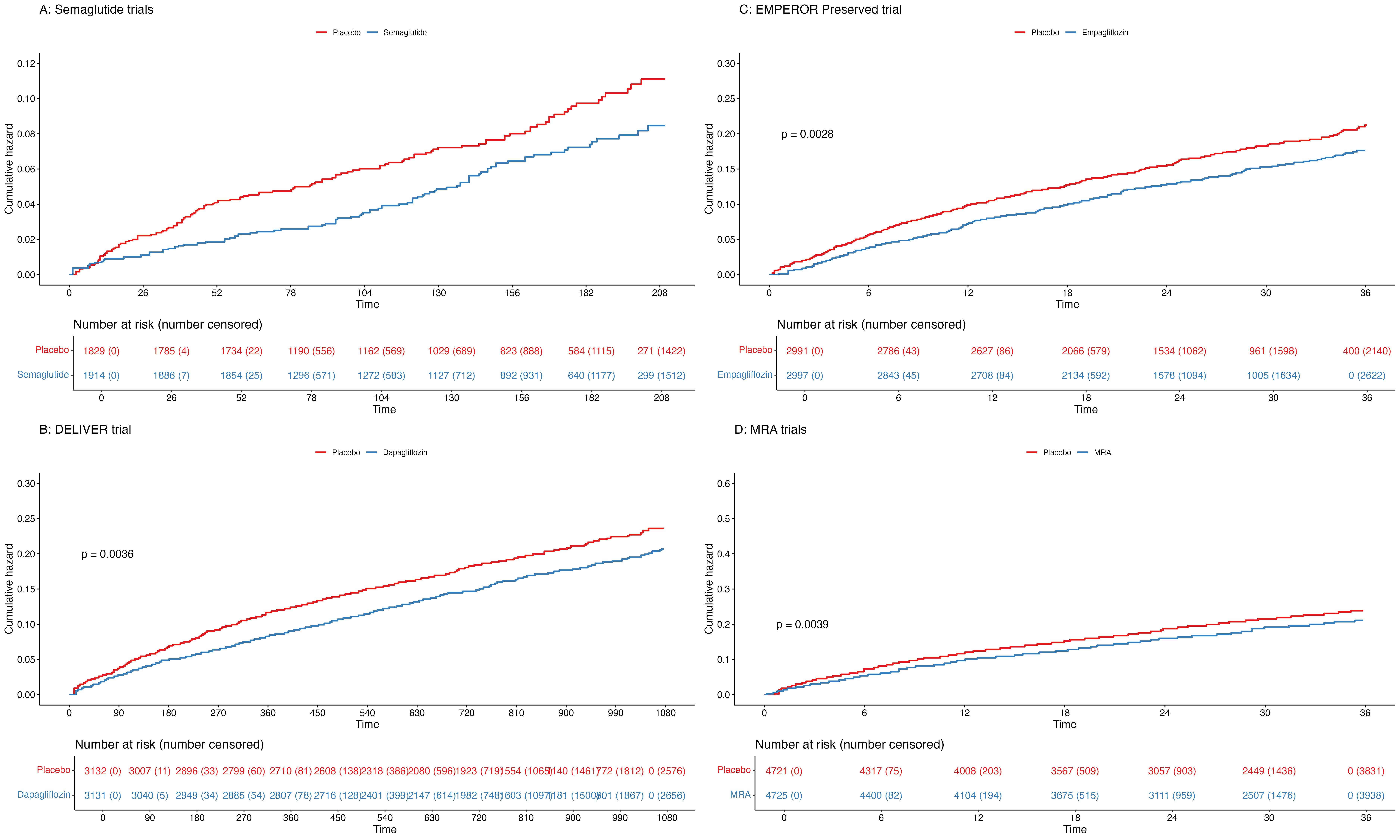
**

MRA: steroid and non-steroid mineralocorticoid receptor antagonists; **DELIVER:** Dapagliflozin Evaluation to Improve the Lives of Patients with Preserved Ejection Fraction Heart Failure; **EMPEROR-PRESERVED**: Empagliflozin Outcome Trial in Patients with Chronic Heart Failure with Preserved Ejection Fraction; **TOPCAT**: Treatment of Preserved Cardiac Function Heart Failure with an Aldosterone Antagonist; **FINEARTS-HF**: Finerenone Trial to Investigate Efficacy and Safety Superior to Placebo in Patients with Heart Failure; **SELECT**: Semaglutide Effects on Cardiovascular Outcomes in People with Overweight or Obesity; **FLOW**: Evaluate Renal Function with Semaglutide Once Weekly; **STEP-HFpEF**: Once Weekly on Function and Symptoms in Subjects with Obesity-related Heart Failure with Preserved Ejection Fraction; and **STEP-HFpEF DM**: Semaglutide Treatment Effect in People with Obesity and Heart Failure with Preserved Ejection Fraction and Diabetes Mellitus.

**eFigure S6: reconstructed time-to HF hospitalization for individual included trials**


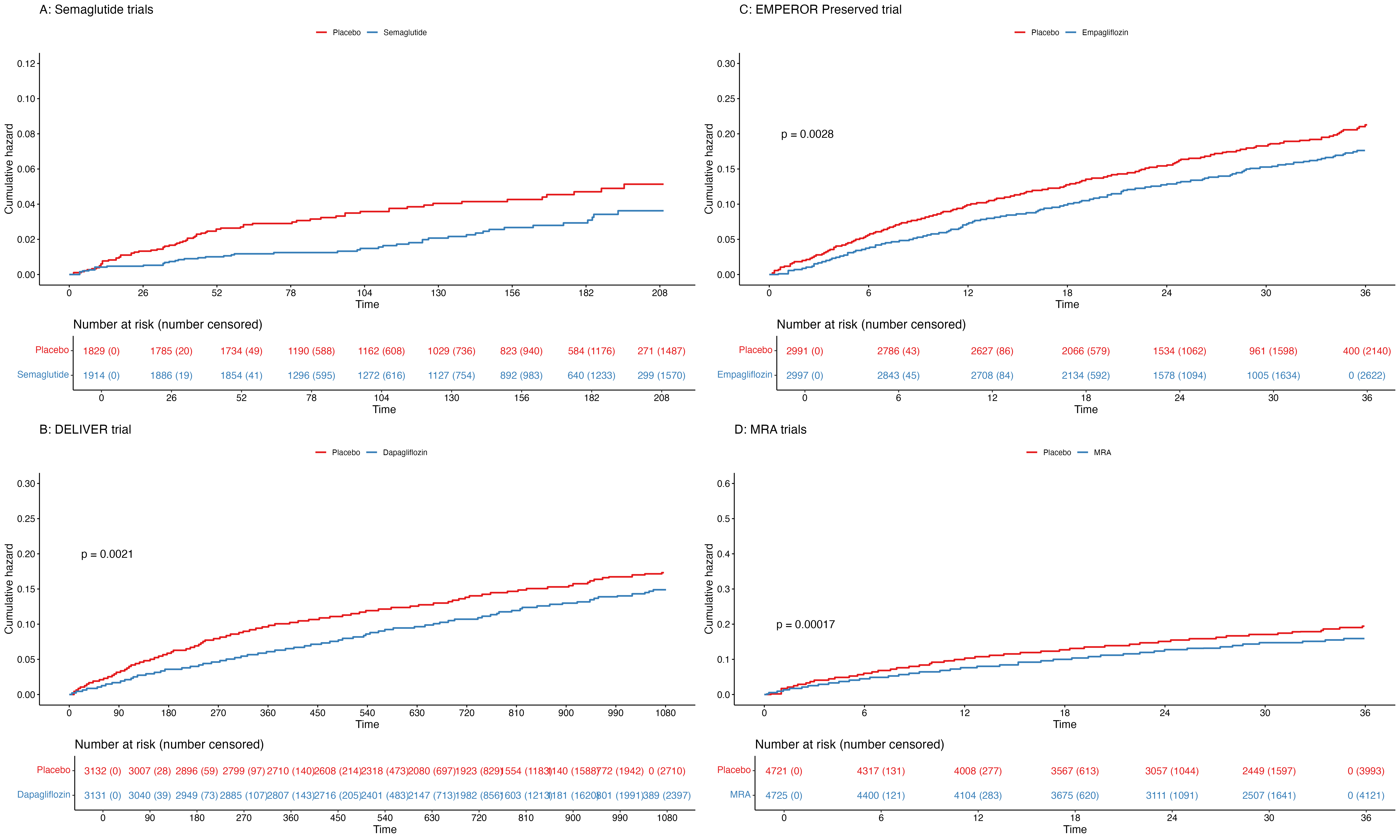


MRA: steroid and non-steroid mineralocorticoid receptor antagonists; **DELIVER:** Dapagliflozin Evaluation to Improve the Lives of Patients with Preserved Ejection Fraction Heart Failure; **EMPEROR-PRESERVED**: Empagliflozin Outcome Trial in Patients with Chronic Heart Failure with Preserved Ejection Fraction; **TOPCAT**: Treatment of Preserved Cardiac Function Heart Failure with an Aldosterone Antagonist; **FINEARTS-HF**: Finerenone Trial to Investigate Efficacy and Safety Superior to Placebo in Patients with Heart Failure; **SELECT**: Semaglutide Effects on Cardiovascular Outcomes in People with Overweight or Obesity; **FLOW**: Evaluate Renal Function with Semaglutide Once Weekly; **STEP-HFpEF**: Once Weekly on Function and Symptoms in Subjects with Obesity-related Heart Failure with Preserved Ejection Fraction; and **STEP-HFpEF DM**: Semaglutide Treatment Effect in People with Obesity and Heart Failure with Preserved Ejection Fraction and Diabetes Mellitus.

**eFigure S7: reconstructed time-to CV death for individual included trials**


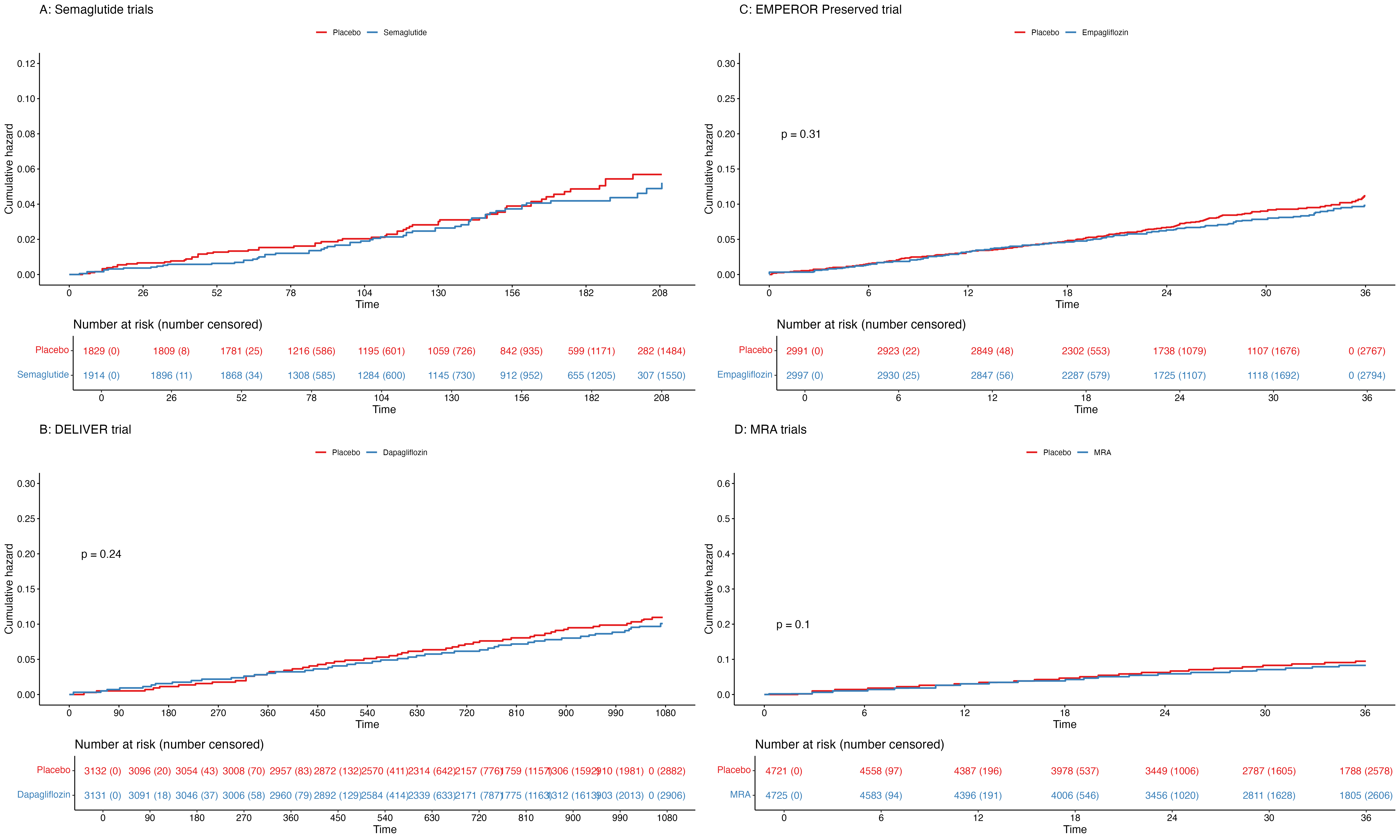


MRA: steroid and non-steroid mineralocorticoid receptor antagonists; **DELIVER:** Dapagliflozin Evaluation to Improve the Lives of Patients with Preserved Ejection Fraction Heart Failure; **EMPEROR-PRESERVED**: Empagliflozin Outcome Trial in Patients with Chronic Heart Failure with Preserved Ejection Fraction; **TOPCAT**: Treatment of Preserved Cardiac Function Heart Failure with an Aldosterone Antagonist; **FINEARTS-HF**: Finerenone Trial to Investigate Efficacy and Safety Superior to Placebo in Patients with Heart Failure; **SELECT**: Semaglutide Effects on Cardiovascular Outcomes in People with Overweight or Obesity; **FLOW**: Evaluate Renal Function with Semaglutide Once Weekly; **STEP-HFpEF**: Once Weekly on Function and Symptoms in Subjects with Obesity-related Heart Failure with Preserved Ejection Fraction; and **STEP-HFpEF DM**: Semaglutide Treatment Effect in People with Obesity and Heart Failure with Preserved Ejection Fraction and Diabetes Mellitus.

**Table S5: Serious adverse events in cardiometabolic included trials**

|  | SGLT2 inhibitors trials | | | | GLP-1 RA trials | | Steroid and non-steroid MRA trials | | | |
| --- | --- | --- | --- | --- | --- | --- | --- | --- | --- | --- |
|  | DELIVER | | EMPEROR-Preserved | | SELECT, FLOW, STEP-HFpEF, and STEP-HFpEF DM | | TOPCAT | | FINEARTS-HF | |
|  | Dapagliflozin (n=3126) | Placebo (n=3127) | Empagliflozin (n=2996) | Placebo (n=2989) | Semaglutide (n=1914) | Placebo (n=1829) | Spironolactone (n=1722) | Placebo (n=1723) | Finerenone (n=3003) | Placebo (n=2998) |
| Any serious adverse events (SAEs) | 1361 (43.5%) | 1423 (45.5%) | 1436 (47.9%) | 1543 (51.6%) | 572 (29.9%) | 708 (38.7%) | 835 (48.5%) | 855 (49.6%) | 1157 (38.5%) | 1213 (40.5%) |
| SAEs leading to treatment discontinuation | 182 (5.8%) | 181 (5.8%) | 571 (19.1%) | 551 (18.4%) | 142 (7.4%) | 175 (9.6%) | 283 (16.4%) | 143 (8.2%) | 611 (20.3%) | 616 (20.5%) |

We did not perform directedly adverse events comparison or meta-analyzed because of differential data capture and exact definitions of these safety events in both trials. The safety analyses were done in treated patients who received at least a single dose of the study medication in both trials.

MRA: steroid and non-steroid mineralocorticoid receptor antagonists; SGLT2i: sodium glucose cotransporter 2 inhibitors; GLP-1 RA: glucagon-like peptide 1 receptor agonists; HF: Heart failure; NR: not reported; ARNI: angiotensin-receptor neprilysin inhibitor; BMI: body mass index; LVEF: left ventricular ejection fraction; **DELIVER:** Dapagliflozin Evaluation to Improve the Lives of Patients with Preserved Ejection Fraction Heart Failure; **EMPEROR-PRESERVED**: Empagliflozin Outcome Trial in Patients with Chronic Heart Failure with Preserved Ejection Fraction; **TOPCAT**: Treatment of Preserved Cardiac Function Heart Failure with an Aldosterone Antagonist; **FINEARTS-HF**: Finerenone Trial to Investigate Efficacy and Safety Superior to Placebo in Patients with Heart Failure; **SELECT**: Semaglutide Effects on Cardiovascular Outcomes in People with Overweight or Obesity; **FLOW**: Evaluate Renal Function with Semaglutide Once Weekly; **STEP-HFpEF**: Once Weekly on Function and Symptoms in Subjects with Obesity-related Heart Failure with Preserved Ejection Fraction; and **STEP-HFpEF DM**: Semaglutide Treatment Effect in People with Obesity and Heart Failure with Preserved Ejection Fraction and Diabetes Mellitus.

**Sensitivity analyses: by excluding the comparison of the combination of MRA+SGLT2 vs GLP-1 RA**

**eFigure S8: Network graph of eligible heart failure with mildly reduced or preserved ejection fraction treatment comparison for efficacy.** Line width is proportional to the inverse variance of treatment effect size comparing every pair of treatment. The size of circle is proportional to the number of comparisons. MRA: steroid and non-steroid mineralocorticoid receptor antagonists; SGLT2i: sodium glucose cotransporter 2 inhibitors; GLP-1 RA: glucagon-like peptide 1 receptor agonists.

**
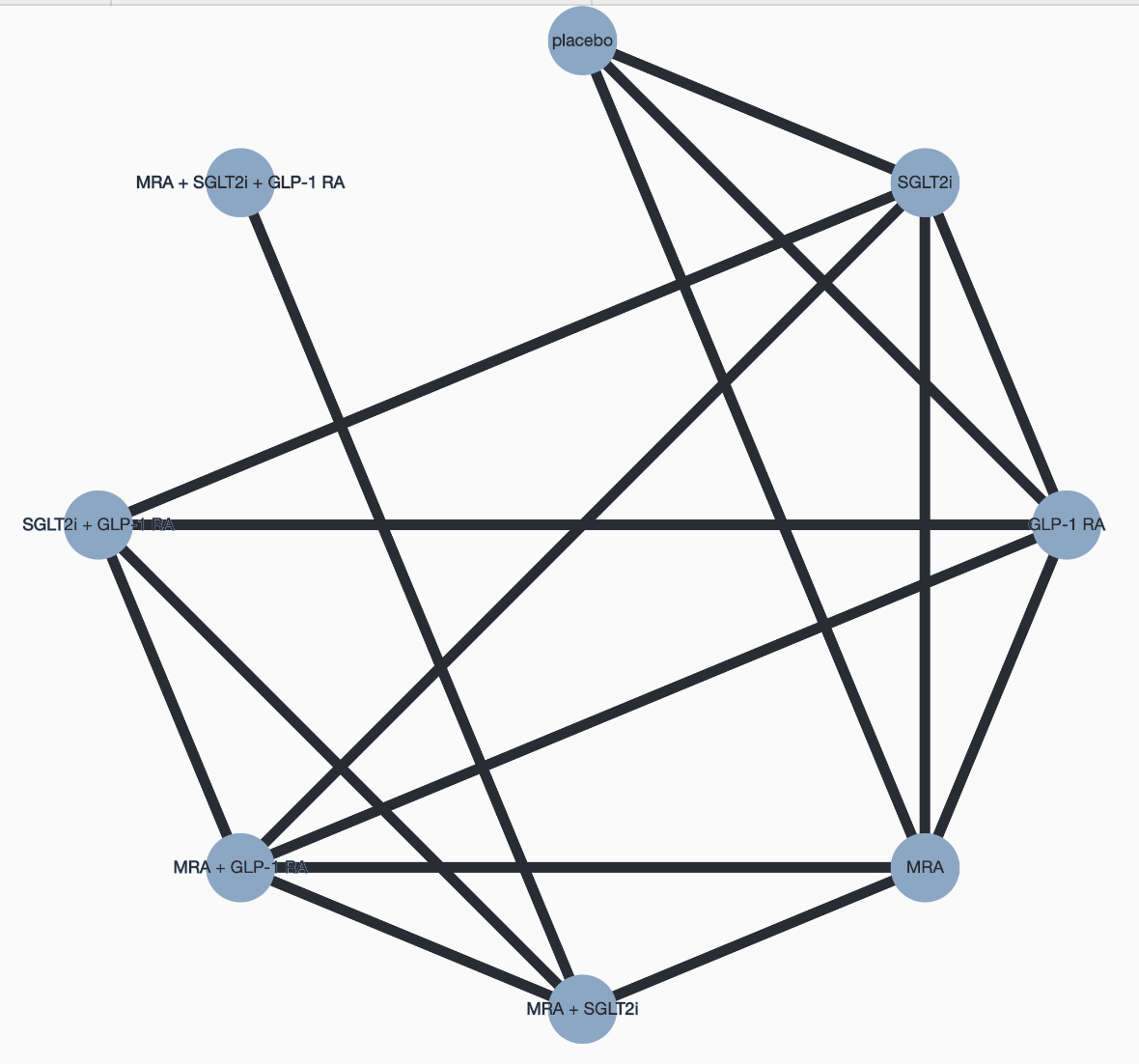
**

**eFigure S9: The quality of evidence by the Confidence in Network Meta-analysis (CINeMA) criteria for a composite of CV death or HF hospitalization**

**eFigure S10: The quality of evidence by the Confidence in Network Meta-analysis (CINeMA) criteria for a HF hospitalization**

**eTable S6: Ligue table from standard network meta-analysis of composite of CV death or HF hospitalization**

| **MRA + SGLT2i + GLP-1 RA** |  |  | |  | |  | |  |  |  |
| --- | --- | --- | --- | --- | --- | --- | --- | --- | --- | --- |
| **0.50 (0.36-0.69)** | **MRA + SGLT2i** | |  |  | |  | |  |  |  |
| **0.47 (0.32-0.70)** | 0.94 (0.75-1.17) | | **SGLT2i + GLP-1 RA** | |  |  | |  |  |  |
| **0.45 (0.31-0.66)** | 0.90 (0.74-1.10) | | 0.96 (0.77-1.20) | | **MRA + GLP-1 RA** | |  |  |  |  |
| **0.34 (0.23-0.48)** | **0.67 (0.57-0.79)** | | **0.72 (0.58-0.88)** | | **0.75 (0.62-0.89)** | | **MRA** |  |  |  |
| **0.33 (0.22-0.49)** | **0.66 (0.53-0.82)** | | **0.71 (0.57-0.88)** | | **0.74 (0.60-0.90)** | | 0.99 (0.83-1.17) | **GLP-1 RA** |  |  |
| **0.33 (0.22-0.48)** | **0.66 (0.54-0.79)** | | **0.70 (0.57-0.85)** | | **0.73 (0.61-0.87)** | | 0.98 (0.86-1.11) | 0.99 (0.84-1.17) | **SGLT2i** |  |
| **0.27 (0.19-0.40)** | **0.55 (0.45-0.67)** | | **0.58 (0.47-0.72)** | | **0.61 (0.50-0.74)** | | **0.81 (0.71-0.93)** | **0.82 (0.69-0.98)** | **0.83 (0.73-0.95)** | **Placebo** |

**eTable S7: Ligue table from standard network meta-analysis of HF hospitalization**

| **MRA + SGLT2i + GLP-1 RA** | |  |  | |  | |  |  |  |
| --- | --- | --- | --- | --- | --- | --- | --- | --- | --- |
| **0.37 (0.23- 0.60)** | **MRA + SGLT2i** |  |  | |  | |  |  |  |
| **0.32 (0.18- 0.58)** | 0.87 (0.63- 1.20) | **SGLT2i + GLP-1 RA** | |  |  | |  |  |  |
| **0.33 (0.19- 0.58)** | 0.89 (0.67- 1.19) | 1.02 (0.74- 1.42) | | **MRA + GLP-1 RA** | |  |  |  |  |
| **0.21 (0.12- 0.36)** | **0.56 (0.44- 0.71)** | **0.64 (0.47- 0.87)** | | **0.63 (0.48- 0.81)** | | **MRA** |  |  |  |
| **0.20 (0.11- 0.36)** | **0.54 (0.39- 0.74)** | **0.62 (0.45- 0.86)** | | **0.61 (0.45- 0.82)** | | 0.97 (0.75- 1.24) | **GLP-1 RA** |  |  |
| **0.20 (0.11- 0.34)** | **0.53 (0.40- 0.70)** | **0.61 (0.45- 0.82)** | | **0.59 (0.45- 0.78)** | | 0.95 (0.79- 1.14) | 0.98 (0.76- 1.26) | **SGLT2i** |  |
| **0.15 (0.09- 0.27)** | **0.41 (0.31- 0.55)** | **0.47 (0.34- 0.65)** | | **0.46 (0.34- 0.62)** | | **0.74 (0.61- 0.89)** | **0.76 (0.59- 0.99)** | **0.78 (0.64- 0.94)** | **Placebo** |

MRA: steroid and non-steroid mineralocorticoid receptor antagonists; SGLT2i: sodium glucose cotransporter 2 inhibitors; GLP-1 RA: glucagon-like peptide 1 receptor agonists; HF: Heart failure; CV: cardiovascular; HR: hazard ratio; CI: confidence interval; value in bracket is the P-score ranking value; bold value is significant comparisons.

**eTable S8: Ranking P-score value for clinical outcomes from additive network meta-analysis**

| **Comparison** | **P score** |
| --- | --- |
| **A composite of CV death or HF hospitalization** |  |
| MRA + SGLT2i + GLP-1 RA | 1.0000 |
| SGLT2i + GLP-1 RA | 0.7310 |
| MRA + GLP-1 RA | 0.7145 |
| MRA + SGLT2i | 0.6965 |
| GLP-1 RA | 0.3035 |
| SGLT2i | 0.2855 |
| MRA | 0.2690 |
| Placebo | 0.0000 |
| **HF hospitalization** |  |
| MRA + SGLT2i + GLP-1 RA | 1.0000 |
| MRA + GLP-1 RA | 0.7425 |
| MRA + SGLT2i | 0.7012 |
| GLP-1 RA + SGLT2i | 0.6984 |
| MRA | 0.3016 |
| GLP-1 RA | 0.2988 |
| SGLT2i | 0.2575 |
| Placebo | 0.0000 |

MRA: steroid and non-steroid mineralocorticoid receptor antagonists; SGLT2i: sodium glucose cotransporter 2 inhibitors; GLP-1 RA: glucagon-like peptide 1 receptor agonists; HF: Heart failure; CV: cardiovascular; HR: hazard ratio; CI: confidence interval.

**eFigure S11: Combination of treatment-effect on composite of CV death or hospitalization for HF (composite primary outcome) (A), and HF hospitalization (B).** MRA: steroid and non-steroid mineralocorticoid receptor antagonists; SGLT2i: sodium glucose cotransporter 2 inhibitors; GLP-1 RA: glucagon-like peptide 1 receptor agonists; HF: Heart failure; CV: cardiovascular; HR: hazard ratio; CI: confidence interval; CNMA: combined network meta-analysis; NMA: network meta-analysis.


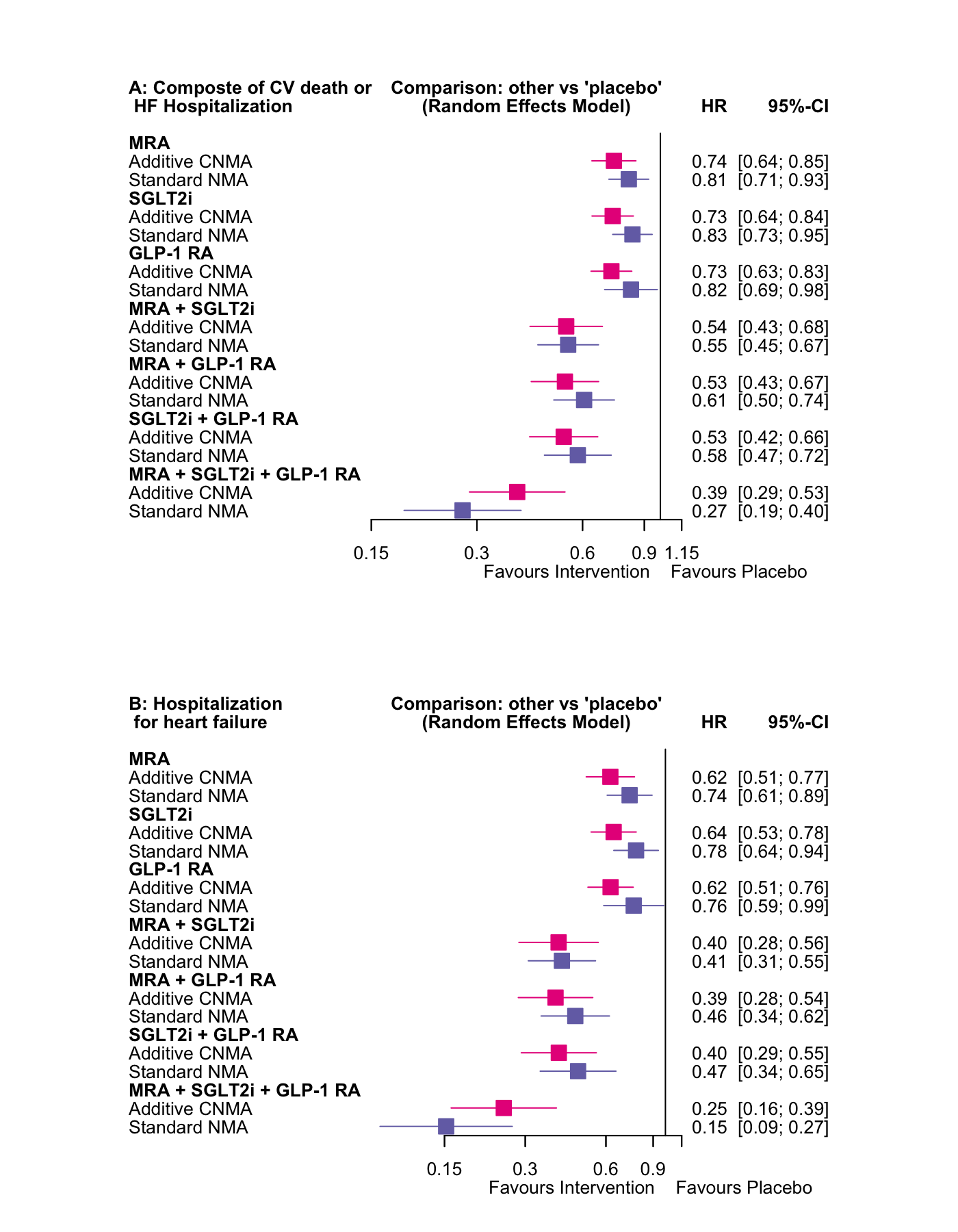

Supplement: Appendix Figs. S1–S11 and Tables S1–S8 [file mmc1.docx]
